# Novel, low-cost solid-liquid-solid process for the synthesis of α-Si_3_N_4_ nanowires at lower temperatures and their luminescence properties

**DOI:** 10.1038/srep17250

**Published:** 2015-11-26

**Authors:** Haitao Liu, Zhaohui Huang, Juntong Huang, Minghao Fang, Yan-gai Liu, Xiaowen Wu, Xiaozhi Hu, Shaowei Zhang

**Affiliations:** 1 School of Materials Science and Technology, Beijing Key Laboratory of Materials Utilization of Nonmetallic Minerals and Solid Wastes, National Laboratory of Mineral Materials, China University of Geosciences (Beijing), 100083, P. R. China; 2 College of Engineering, Mathematics and Physical Sciences, University of Exeter, Exeter EX4 4QF, UK; 3 Department of Mechanical Engineering, University College London, Torrington Place, London WC1E 7JE, UK; 4 School of Mechanical and Chemical Engineering, The University of Western Australia, Australia

## Abstract

Ultra-long, single crystal, α-Si_3_N_4_ nanowires sheathed with amorphous silicon oxide were synthesised by an improved, simplified solid-liquid-solid (SLS) method at 1150 °C without using flowing gases (N_2_, CH_4_, Ar, NH_3_, *etc.*). Phases, chemical composition, and structural characterisation using X-ray diffraction (XRD), field emission scanning electron microscopy (FESEM), transmission electron microscopy (TEM/HRTEM), Fourier-transform infrared spectroscopy (FT-IR), and X-ray photoelectron spectroscopy (XPS) showed that the nanowires had Si_3_N_4_@SiO_*x*_ core-shell structures. The growth of the nanowires was governed by the solid-liquid-solid (SLS) mechanism. The room temperature photoluminescence (PL) and cathodoluminescence (CL) spectra showed that the optical properties of the α-Si_3_N_4_ nanowires can be changed along with the excitation wavelength or the excitation light source. This work can be useful, not only for simplifying the design and synthesis of Si-related nanostructures, but also for developing new generation nanodevices with changeable photoelectronic properties.

One-dimensional (1-d) nanostructures have been observed in a number of material systems^1^,^2^,^3^,^4^. Researchers have studied 1-d nanostructured materials due to their remarkable properties (including optical, electronic, mechanical properties, *etc.*) and significant potential applications in optoelectronic devices have been found^5^,^6^,^7^. In recent years, various 1-d nanomaterials have been fabricated, such as: C nanotubes^8^,^9^, Ag nanowires^10^, Cu nanowires^11^, Si nanowires^12^,^13^, SiC nanochains^14^, Si_3_N_4_ nanobelts^15^, ZnO nanosprings^16^, ZnO nanohelices^17^, *etc.*

Silicon nitride (Si_3_N_4_), an important wide-band gap semiconducting material with several applications in optoelectronics, can form single crystal nanowires, nanobelts, nanodendrites, and nanosheets when treated with different methods^18^,^19^,^20^,^21^. Traditionally, 1-d Si_3_N_4_ nanomaterials are prepared by chemical vapour deposition (CVD)^22^, plasma enhanced CVD (PECVD)^23^, microwave plasma heating^24^,^25^, catalytic pyrolysis of a polymer precursor^26^, sol-gel^27^, combustion synthesis^28^, *etc.* The CVD method is considered to be a potential method for the synthesis of Si_3_N_4_ nanostructures due to its low cost and simplicity. Lin *et al.*^22^ prepared large-scale α-Si_3_N_4_ nanowires by a catalyst-free CVD route at 1400–1550 °C using N_2_ as the nitrogen source, SiO, or a mixture of Si and SiO_2_, as the silicon source, N_2_ and Ar as the barrier gas, and CH_4_ as the reducing gas. Huang *et al.*^29^ synthesised ultra-long, single crystal, α-Si_3_N_4_ nanobelts on graphite deposited with Ni(NO_3_)_2_ by CVD method with flowing N_2_ at 1450 °C. Huo *et al.*^30^ developed a CVD method for the production of single-crystalline α-Si_3_N_4_ nanobelts, consisting of the nitridation of a high Si content Fe-Si catalyst by NH_3_ at 1300 °C. However, most of these CVD processes involved complicated equipment, vacuum conditions, and relatively high temperatures. These issues limit the production and application of Si_3_N_4_ nanostructured materials. Considering efficiency and economy, a simplified solid-liquid-solid (SLS) process is considered to be a potentional method for the synthesis of Si_3_N_4_ nanowires due to its low energy consumption.

We now report an improved and simplified SLS method without using flowing gases (N_2_, CH_4_, Ar, NH_3_, *etc.*) to synthesise α-Si_3_N_4_ nanowires at the lower temperature of 1150 °C. The resulting α-Si_3_N_4_ nanowires were structurally characterised and found to have tunable luminescent properties, making them attractive for new optoelectronic applications.

## Results and Discussion

**Figure 1:**
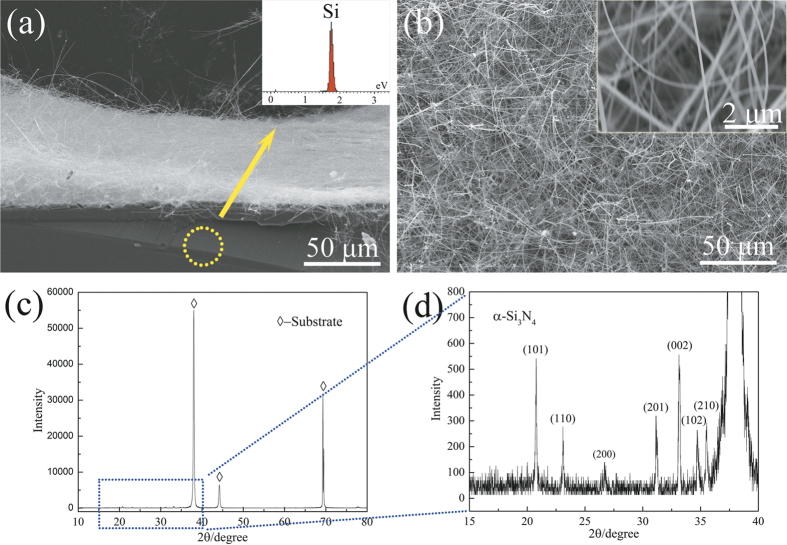
(**a,b**) Representative lower magnification FESEM images of as-grown nanowires found on the substrate. The inset pattern in (**a**) is the EDS spectrum recorded from the marked area in (**a**). The inset image in (**b**) is a higher magnification FESEM image of the products. (**c,d**) X-ray diffraction pattern of the nanowire products covering the substrate.

In this research, an improved, simplified SLS method, without using flowing gases (N_2_, CH_4_, Ar, NH_3_, *etc.*) was used to synthesise Si_3_N_4_ nanowires at a lower temperature. White coloured layers of fluffy materials, extending from the substrate surfaces up to a few microns in height (as shown in Fig. 1a), were visible to the naked eye on the substrates. Low resolution FESEM images showed the high degree of uniformity of the nanowires, which can be as long as several hundred microns. The best nanowire growth in terms of morphology and yield was observed for a substrate heated to 1150 °C. EDS spectroscopy analysis (see inset, Fig. 1a) indicated that the substrate was Si without detectable impurities therein. The inset to Fig. 1b shows a higher magnification FESEM image, revealing that the products were actually nanowires with diameters ranging from 80 to 150 nm. Figure 1c shows the X-ray diffraction (XRD) pattern of the nanowire products covering the substrate. The strong intensities and narrow widths of the peaks indicated that the substrate was well crystallised. The partial, enlarged, view (Fig. 1d) shows that the relatively weak peaks can be matched to those of the α-Si_3_N_4_. Together with the EDS results, and considering the crystallinity of the substrate and nanowires, it was expected that the as-formed nanowires were α-Si_3_N_4_.

**Figure 2:**
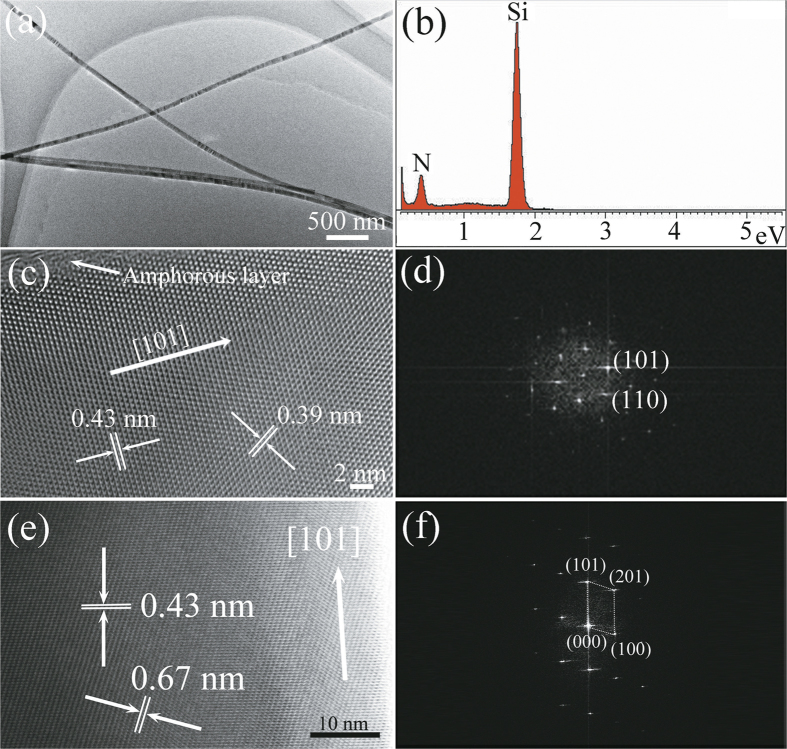
Crystal structure and chemical composition of the nanowires. (**a**) Typical low-magnification TEM image of the α-Si_3_N_4_ nanowires, showing its structural uniformity. (**b**) The corresponding EDS spectrum recorded from the single nanowire. (**c,e**) High-magnification TEM images of single nanowires and (**d,f**) their corresponding fast Fourier transform (FFT) pattern.

The α-Si_3_N_4_ nanowires were further characterised using transmission electron microscopy (TEM). The as-prepared nanowires were sonicated and suspended in ethanol, dispersed onto a TEM micro-grid with a lace-like carbon film, and measured with TEM/HRTEM equipped with an EDS detector. A representative low-magnification TEM image of typical nanowires (Fig. 2a) shows that a single nanowire had a uniform diameter. High-resolution TEM (HRTEM) images (Fig. 2c,e) of the representative α-Si_3_N_4_ nanowires show lattice fringes of a single crystal nanowire. The chemical composition of a single nanowire was analysed by EDS. As shown in Fig. 2b, the EDS spectrum revealed that the nanowire consisted of Si and N elements. The measured lattice spacings observed in HRTEM were 0.67 nm, 0.43 nm, and 0.39 nm, corresponding well to the (100), (101), and (110) lattice spacings of the α-Si_3_N_4_ structure, respectively. The 2-d fast Fourier transform (FFT) of the lattice resolved images (Fig. 2d,f) shows the reciprocal lattice peaks, which can be indexed to a primitive hexagonal lattice. Indexed reciprocal lattices observed showed that the nanowires closely matched the structure of α-Si_3_N_4_. The HRTEM image in Fig. 2c shows an amorphous phase in the outer shell region. In this research, before the temperature was increased, there were still unexpelled gases remaining in the reaction system (including oxygen). This suggested that the amorphous shell may be formed during the high temperature nanowire synthesis process, in which a trace amount of oxygen could be present in the simplified SLS set-up used here. It is generally understood that surface oxidation of silicon-related products generates silicon oxide^31^. In this research, this was further investigated by FT-IR and XPS.

**Figure 3:**
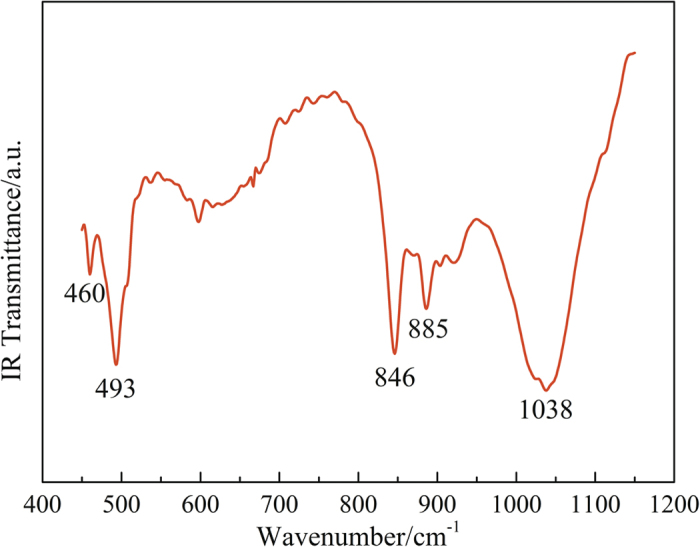
FT-IR absorption spectrum of the as-prepared products.

Figure 3 shows the absorption FT-IR spectra of the α-Si_3_N_4_ nanowires. The broad spectrum was taken over the range 450–1150 cm^−1^ with a step size of 1 cm^−1^. The bonding signatures of the as-prepared nanowires showed five distinguishing vibrational bands: the vibrational bands at 800–1100 cm^−1^ (846 cm^−1^, 885 cm^−1^, and 1038 cm^−1^) were derived from the Si-N stretching vibration mode of α-Si_3_N_4_^32^. The absorption peak at 460 cm^−1^ corresponded to Si-N stretching in β-Si_3_N_4_^33^,^34^, suggesting that there may be a little β-Si_3_N_4_ in the as-synthesised products. The peak at 493 cm^−1^ was due to the Si-O-Si stretching vibration of amorphous SiO_*x*_^35^.

**Figure 4:**
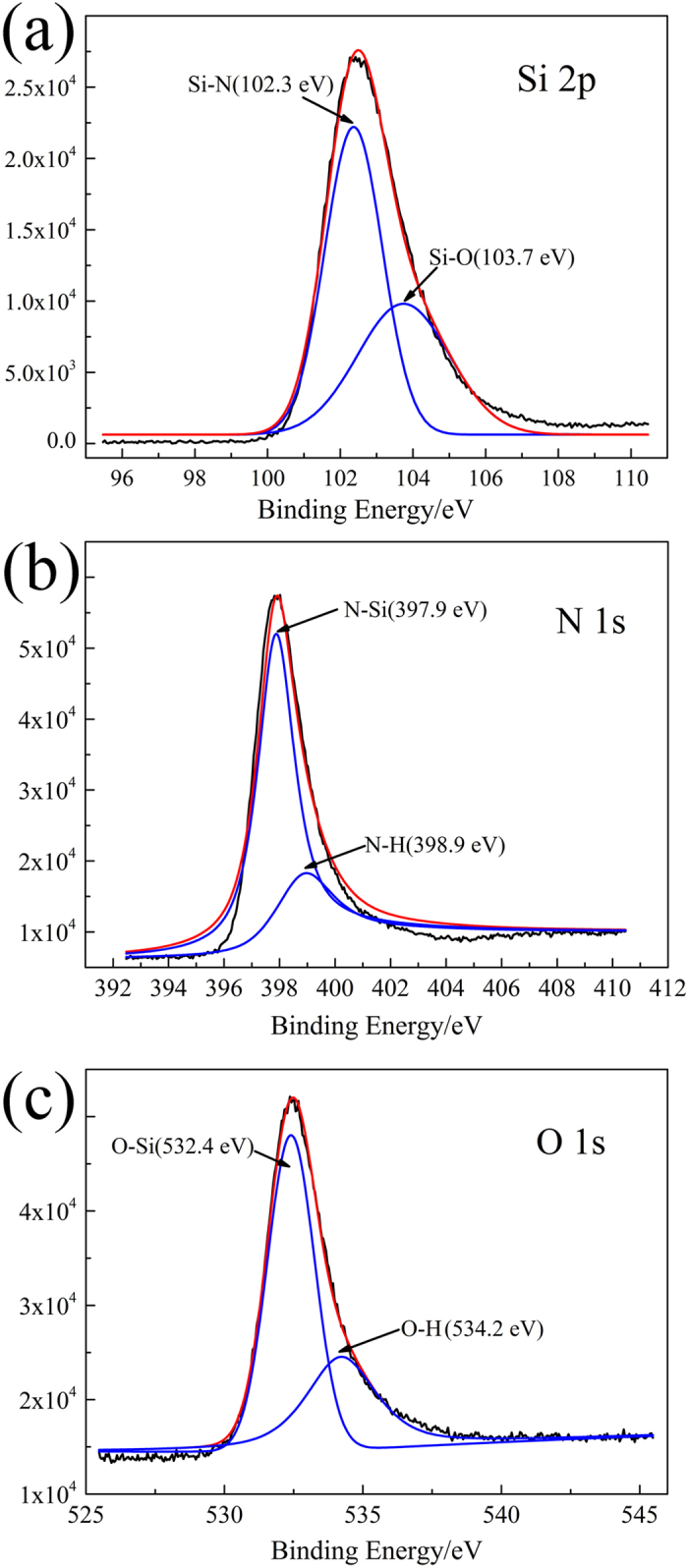
XPS spectra of the as-synthesized α-Si_3_N_4_ nanowires: (a) Si 2p; (b) N 1s; (c) O 1s.

To confirm further the structure of these nanowires, XPS spectroscopy was used to verify the bonding structure thereof. Figure 4 shows the core-level XPS spectra of the Si 2p, N 1s, and O 1s regions of the α-Si_3_N_4_ nanowires synthesised by the improved, simplified SLS method. All of the signal curves were fitted by the Gaussian method. A Shirley fitting procedure was used to determine the background to each spectrum and a Gaussian-Lorentzian peak shape with a G/L ratio of 80 was adopted. Figure 4a shows that the binding energy of the Si 2p core orbital spectra lay within the range 95–115 eV. The peak energy associated with 102.3 eV corresponded to the Si-N bond, and the peak energy located at 103.7 eV was attributed to the Si-O bond which is associated with the outer oxide layer^22^. The resolved component of N 1s is shown in Fig. 4b. The peak at 397.9 eV corresponded to the binding energy of the N-Si bond^36^. The binding energy at around 398.9 eV could be attributed to the N-H on the surface^37^. In addition to Si 2p, and N 1s, the XPS spectra of O 1s are also measured as shown in Fig. 4c. From the XPS analysis, the peaks located at 532.4 eV and 534.2 eV corresponded to the binding energy of O-Si and O-H bonds, respectively^38^. The O 1s peak was related to the presence of SiO_*x*_ and the O-H came from the surface oxidation layer. The values of Si 2p, N 1s, and O 1s peaks were close to those for Si_3_N_4_ and SiO_*x*_, which demonstrated that the chemical bonds of Si-N and Si-O had formed in the α-Si_3_N_4_ nanowires. Based on the XRD, TEM, FT-IR, and XPS, results it could be concluded that the as-prepared nanowires consisted mainly of α-Si_3_N_4_ wrapped with an amorphous SiO_*x*_ shell.

In recent years, 1-d nanostructures are usually synthesised by vapour-solid (VS), vapour-liquid-solid (VLS), or solid-liquid-solid (SLS) mechanisms, depending on the absence, or presence, of metal catalysts or gases in the preparation process. Among these methods, the SLS process seems to be an optimal method for generating 1-d nanostructures at lower temperatures. Nanowires are conventionally assumed to grow by SLS procedure, in which material arising from the solid is incorporated by a liquid catalyst, commonly a low-melting point eutectic alloy^39^.

**Figure 5:**
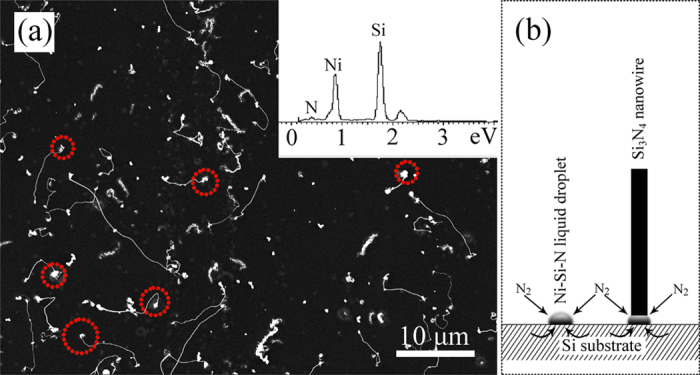
(**a**) A typical FESEM image of the α-Si_3_N_4_ nanowires prepared at 1100 ^°^C, the inset pattern is the EDS spectrum recorded from the top of a single nanowire marked by the dotted rings. (**b**) Schematic illustration for the growth of α-Si_3_N_4_ nanowires.

In order to investigate the growth mechanism of the α-Si_3_N_4_ nanowires, as shown in Fig. 5a, numbers of nanowires products were also obtained when sintering at 1100 °C. The inset in Fig. 5a shows the EDS spectrum recorded from the bottom of a single nanowire marked by the dotted rings. As can be seen, a number of apparent catalytic base exist at the bottom of the nanowires: EDS analysis showed that the catalytic dots mainly contained Si, Ni, and N elements, which indicated that the presence of minute amounts of Ni(NO_3_)_2_ was crucial to the formation of a large amount of such nanowires. It was believable that nanowire growth stemmed from a SLS mechanism. On account of the eutectic point of NiSi_2_ is 993 °C (which can be seen from the Si-Ni diagram)^40^, Si atoms diffused into Ni-Si alloy droplets continuously. The N_2_ vapour phase diffused into the Ni-Si alloy particles, forming Ni-Si-N liquid droplets. Based on the aforementioned processes, Si_3_N_4_ nuclei and nanowires were generated on the Si substrate when the droplet reached supersaturation. Figure 5b shows the proposed growth process of the α-Si_3_N_4_ nanowires. The trace amount of Ni (decomposed from Ni(NO_3_)_2_) provided initial nucleation sites for the formation of Ni-Si-N liquid droplets.

**Figure 6:**
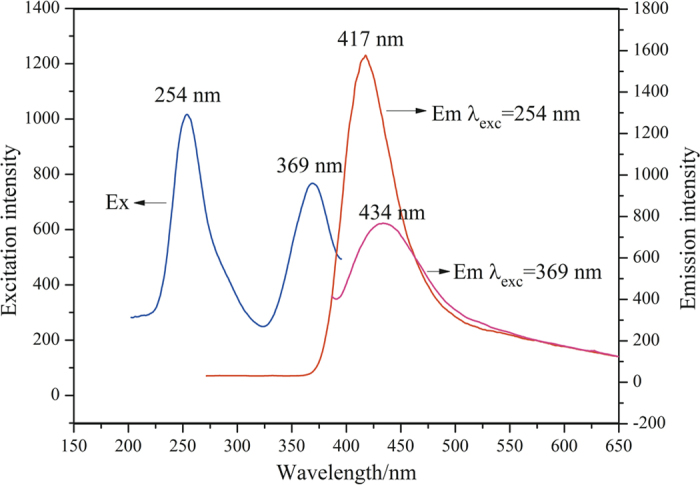
The excitation and emission spectra of α-Si_3_N_4_ nanowires. The excitation spectrum monitored at 417 nm indicated two excitation peaks at 254 nm and 369 nm. The emission spectra were measured under 254 nm and 369 nm excitation, respectively.

To investigate the optical properties of the as-synthesised α-Si_3_N_4_ nanowires, their PL and CL spectra were recorded at room temperature. Figure 6 shows the room temperature PL spectra of the α-Si_3_N_4_ nanowires. The excitation spectrum was taken over the range 200 to 400 nm, and was monitored at 417 nm (2.97 eV). The excitation signatures of the as-synthesised α-Si_3_N_4_ nanowires showed two distinct peaks centred around 254 nm (4.88 eV) and 369 nm (3.36 eV), respectively. Therefore, the emission spectra were recorded under these two excitation wavelengths. The results showed that the PL intensity excited at 254 nm (4.88 eV) was much higher than that excited at 369 nm (3.36 eV), and the peak position was slightly red-shifted with decreasing excitation wavelength. Strong emission spectra were located in the violet-blue spectral range, centred around 417 nm (2.97 eV) and 434 nm (2.86 eV), respectively, when excited by light with wavelengths of 254 nm (4.88 eV) and 369 nm (3.36 eV). The two emission bands were both considerably red-shifted, compared with the direct band gap of α-Si_3_N_4_ (5.2 to 5.3 eV)^22^,^30^. These PL results were different from those in previous reports^29^,^41^. Robertson *et al.* define four types of defects in Si_3_N_4_, including: Si-Si, N-N, =N^0^, and ≡Si^0^ dangling bonds. As previously reported^29^,^42^, the luminescence centred at approximately 417 nm (2.97 eV) should arise due to recombination, either from the conduction band to the N_2_^0^ level, or the valence band to the N_4_^+^ level. As proposed, an amorphous oxide layer existed on the surface of the α-Si_3_N_4_ nanowires, as determined by TEM/HRTEM, FT-IR, and XPS. As previously reported, the emission bands centred around 434 nm (2.86 eV) could arise from the electronic transitions from ≡Si^0^ to N-Si-O^29^,^30^,^43^. Based on previous research, in the current results, when excited by 254 nm (4.88 eV) wavelength light, we believed that the recombination from the conduction band to the N_2_^0^ level or the valence band to the N_4_^+^ level dominated the emission properties, together with the electronic transitions from ≡Si^0^ to N-Si-O. Nevertheless, the electronic transitions from ≡Si^0^ to N-Si-O dominated the emission properties when excited by light with a wavelength of 369 nm (3.36 eV).

**Figure 7:**
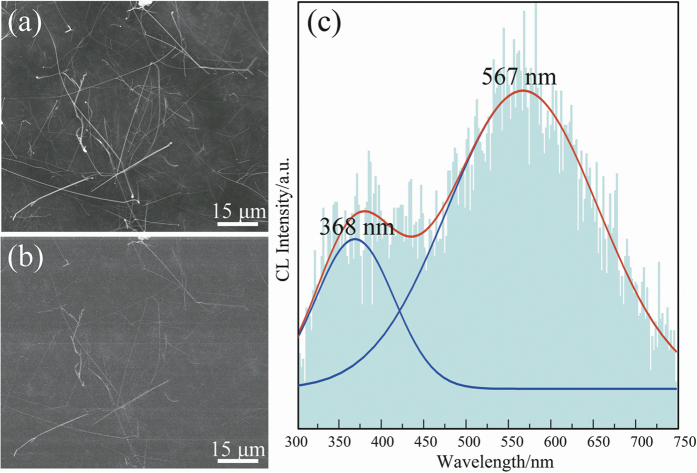
(**a–b**) SE and CL images of the α-Si_3_N_4_ nanowires; (**c**) Room-temperature CL spectra of α-Si_3_N_4_ nanowires with a focused electron beam at an accelerating voltage of 30 kV.

To visualise the spatial distribution of the luminescence from these α-Si_3_N_4_ nanowires, the secondary electron (SE) image and corresponding CL image were recorded as shown in Fig. 7a–b. Figure 7c shows the room temperature CL spectrum of the products. As shown in Fig. 7c, the histogram is the as-obtained spectrum, and the red and blue lines are simulated data plots. The nanowire spectra showed two peaks at approximately 368 nm (3.37 eV) and 567 nm (2.19 eV). Previous studies suggested that the optical properties of nanostructured materials can be affected by many factors, such as intrinsic characteristics, composition, shape and size of nanomaterial, structural defects, and impurities^44^,^45^. Hu *et al.*^46^ reported the CL spectrum of an α-Si_3_N_4_ microribbon, which consists of one intense UV emission peak at approximately 305 nm (4.06 eV) and two weak broad peaks at around 540 nm (2.30 eV) and 735 nm (1.68 eV). They considered the 305 nm peak as being due to recombination, either from the conduction band to the N_2_^0^ level, or from the valance band to the N_4_^+^ level: the 540 nm peak is attributed to a recombination process at the silicon dangling bond, and the 735 nm peak is caused by recombination between the N_4_^+^ and N_2_^0^ levels^46^. Huang *et al.*^47^ synthesised α-Si_3_N_4_ nanobelts, nanowires, and nanobranches and compared the CL properties of these three nanostructures. They propose that the UV-blue emissions of their α-Si_3_N_4_ nanobelt, nanowire, and nanobranch centred with a band from 3.05 eV to 3.34 eV should arise from a recombination between the Si-Si σ^*^ level and the N_2_^0^, and N_2_^0^, levels, or between the N_4_^+^ and intrinsic valence band edge^47^.

In this research, under an accelerating voltage of 30 kV, the electrons could be injected to a depth of hundreds of nanometres, or even several microns, into the samples. This may have caused a different dominant luminescence mechanism between PL and CL measurements. The CL emission of the α-Si_3_N_4_ nanowires centred at approximately 368 nm (3.37 eV) should arise from a recombination between the Si-Si σ^*^ level and the N_2_^0^ level^47^. The peak at approximately 567 nm (2.19 eV) was ascribed to the recombination process at the silicon dangling bond^46^. From the PL and CL results, it can be concluded that the luminescence property of the α-Si_3_N_4_ nanowires can be changed along with the excitation wavelength or the excitation light source. The changeable emission properties of the α-Si_3_N_4_ nanowires synthesised by our simplified SLS method are of significant interest for their potential application in new photoelectric nanodevices.

## Conclusions

Ultra-long, single-crystal, α-Si_3_N_4_ nanowires have been obtained by using an improved, simplified, solid-liquid-solid method at 1150 °C. The as-synthesised nanowires were up to several hundred microns in length. Furthermore, the resulting α-Si_3_N_4_ nanowires were structurally characterised and found to have Si_3_N_4_@SiO_*x*_ core-shell nanostructures with a single crystal Si_3_N_4_ core and an insulating amorphous silicon oxide shell. The formation process was considered to be dominated by a solid-liquid-solid mechanism. The room temperature photoluminescence and cathodoluminescence spectra indicated that the luminescence of the α-Si_3_N_4_ nanowires could be changed along with the excitation wavelength or the excitation light source. The data presented here could also be helpful in simplifying the design and synthesis of Si-related nanostructures. Furthermore, the changeable luminescence properties can also be applied to new generation nanodevices with tunable photoelectronic properties.

## Methods

**Figure 8:**
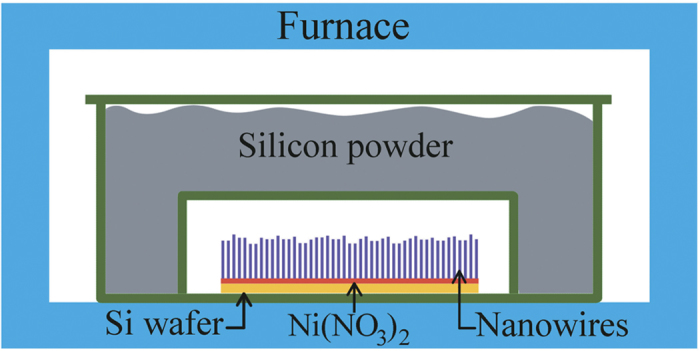
Schematic diagram of experimental setup for α-Si_3_N_4_ nanowires synthesis.

Si_3_N_4_ nanowires were prepared, with high reproducibility, by a SLS process, without using flowing gases (N_2_, CH_4_, Ar, NH_3_, *etc.*). The simple experimental set-up consisted of a horizontal high-temperature tube furnace and two aluminium alloy crucibles (as shown in Fig. 8). First, an n-type Si (100) wafer (4 Ω·cm, Beijing Zhongkekenuo New Energy Technology Co. Ltd, China) was ultrasonically cleaned in acetone and ethanol for 10 minutes in each solvent, and then dried in air. The cleaned substrate was dipped into 0.01 m nickel nitrate (Ni(NO_3_)_2_) aqueous solution and dried at room temperature. After that, the silicon substrate was loaded into an aluminium alloy boat, and covered by another smaller aluminium alloy boat (25 mL). Then, a corundum boat was filled with metallic silicon powder and covered by a matching corundum lid. Therefore, the Si (100) wafer with its Ni(NO_3_)_2_ catalyst was separated with another Si source (metallic silicon powder). The aforementioned enclosed system was then placed in a furnace and heated in air. The furnace temperature was increased at 10 °C/min from room temperature to 1000 °C, and then increased at 3 °C/min from 1000 °C to 1150 °C, then held at that temperature for 3 h. After cooling to room temperature, a white layer was formed on the surface of the Si substrate. It was worth noting that the metallic silicon powder could be recycled and reused.

The copious amount of nanowires thus produced allowed the identification of phases in the product samples using powder X-ray diffraction (XRD). The morphology and microstructure of the as-grown nanowires were characterised using a variety of microscopes and energy dispersive X-ray spectroscopy (EDS), field emission scanning electron microscopy (FESEM, JEOL JSM6700F, Japan) at 20 kV, and high-resolution transmission electron microscopy (TEM/HRTEM, FEI-Tecnai-G^3^-F20, Philips, Netherlands) at 300 kV. A standard KBr pellet technique was used for further Fourier-transform infrared spectroscopy (FT-IR) examination. The FT-IR spectrum was collected with a Nicolet IR100/200 spectrophotometer over the wavenumber range of 450–1150 cm^−1^. X-ray photoelectron spectroscopy (XPS, ESCALAB 250Xi, Michigan, USA) measurements were performed using an aluminium Kα micro-focused monochromator with a spot size of 500 μm. Total scans of all the core levels (Si(2p), N(1s), and O(1s)) with kinetic energy measurement were realised in 0.05 eV increments. To investigate the optical properties of the as-prepared α-Si_3_N_4_ nanowires, their photoluminescence (PL) and cathodoluminescence (CL) spectra were recorded at room temperature. A fluorescence spectrophotometer (Hitachi F4600, Japan) with a Xe lamp excitation source was used to record the room temperature PL spectra. A high-resolution CL system (an ultra-high vacuum scanning electron microscope (UHV-SEM), equipped with a Gemini electron gun (Omicron, Germany) and a CL detector (Gatan mono 3 plus)) at an accelerating voltage of 30 kV was used to collect the CL spectra. The CL images and spectra were collected at room temperature.

## Additional Information

**How to cite this article**: Liu, H. *et al.* Novel, low-cost solid-liquid-solid process for the synthesis of α-Si_3_N_4_ nanowires at lower temperatures and their luminescence properties. *Sci. Rep.* **5**, 17250; doi: 10.1038/srep17250 (2015).
